# Hemoglobin diffusion and the dynamics of oxygen capture by red blood cells

**DOI:** 10.1038/s41598-017-09146-9

**Published:** 2017-09-05

**Authors:** Stéphane Longeville, Laura-Roxana Stingaciu

**Affiliations:** 1 Laboratoire Léon Brillouin, CEA, CNRS, Université Paris-Saclay, CEA-Saclay, F-91191 Gif-sur-Yvette, France; 2 Juelich Centre for Neutron Science, outstation at SNS, Oak Ridge National Laboratory, Oak Ridge, TN 37831 USA

## Abstract

Translational diffusion of macromolecules in cell is generally assumed to be anomalous due high macromolecular crowding of the milieu. Red blood cells are a special case of cells filled quasi exclusively (95% of the dry weight of the cell) with an almost spherical protein: hemoglobin. Hemoglobin diffusion has since a long time been recognized as facilitating the rate of oxygen diffusion through a solution. We address in this paper the question on how hemoglobin diffusion in the red blood cells can help the oxygen capture at the cell level and hence to improve oxygen transport. We report a measurement by neutron spin echo spectroscopy of the diffusion of hemoglobin in solutions with increasing protein concentration. We show that hemoglobin diffusion in solution can be described as Brownian motion up to physiological concentration and that hemoglobin diffusion in the red blood cells and in solutions at similar concentration are the same. Finally, using a simple model and the concentration dependence of the diffusion of the protein reported here, we show that hemoglobin concentration observed in human red blood cells ($\simeq$330 *g*.*L* ^−1^) corresponds to an optimum for oxygen transport for individuals under strong activity.

## Introduction

Whereas protein diffusion in solution at low concentration can readily and remarkably be described by Brownian motion theory^1, 2^, it is supposed to be highly anomalous in the different cell compartments^3^. The origin of this anomalous behavior is not fully understood but is generally predominantly ascribed to macromolecular crowding^4, 5^. The presence of freely moving macromolecules of different size and shape in the cytoplasm (proteins, ribosomes, RNA…) together with filamentous fixed networks are supposed to be responsible of the strong slowing down of protein diffusion and to the power-law increase of the time dependence of their mean-square displacement. From experimental measurements of labeled protein diffusion in the cytoplasm of living cells It was shown that a cross-over occurs from Brownian to anomalous diffusion at a certain length scale^6^. This cross-over was mainly attributed to the presence of relatively immobile structure, suggesting that mobile macromolecules, although significantly reducing the protein diffusion, would lead to Brownian type motion. Recent experimental and simulation work^7^ has shown that, in colloidal model systems of mobile obstacles, anomalous diffusion could occur depending on the size ratio between the probe and the crowder at very high crowding fraction.

Red Blood Cells (RBCs) are a special class of cells in the sense that they contains almost nothing but a protein, the hemoglobin, a 65 kDa tetrameric close to spherical shape molecule. In mammals, RBCs contains no nucleus or cell organelles and can be viewed as a sack containing hemoglobin at high concentration. The sack being composed of a plasma membrane. Human RBCs have biconcave shape with a diameter of ~8 *μm* and a thickness around 1 *μm*. The physiological role of Hemoglobin is gas transport, and among them *O*_2_ and *CO*_2_ are ubiquitous and probably the most important.

Oxygen uptake by the RBCs in the lungs is a complex molecular process occurring over different length and time scales. RBCs are transported by heart driven blood flux in the capillaries close to the alveoli. Molecular oxygen must diffuse from the alveoli to the interior of the RBCs where it binds to hemoglobin (Hb). One molecule of hemoglobin can bind reversibly 4 *O*_2_ molecules. The Hb affinity for oxygen strongly depends on local oxygen partial pressure (${P}_{{O}_{2}}$). In the capillary lungs, where ${P}_{{O}_{2}}$ is high (typically $\simeq$100 Torr $\simeq$13.1 kPa), almost all Hb molecules are charged by 4 *O*_2_ whereas when ${P}_{{O}_{2}}$ decreases (typically near the muscles where the oxygen demand is high) Hb releases its *O*_2_ molecules.The transfer of *O*_2_ from the alveolar air to hemoglobin has been the subject of numerous theoretical, experimental and simulation works (see ref. 8 and references therein). It is a highly complex phenomenon where molecular oxygen must diffuse through membranes, plasmas and finally inside the RBCs trough the cell membrane where it binds to the protein. Hb diffusion has been recognized as facilitating the rate of oxygen capture and/or release by the RBCs. Protein-facilitated diffusion and its possible implication in oxygen transport mechanism was first recognized by Wittenberg^9^ and Scholander^10^ in both myoglobin and hemoglobin solutions. In ref. 10, it was shown that the oxygen flux going through a solution with hemoglobin was significantly increased with respect to the solution without hemoglobin. Later on, Wittenberg^11^ showed that the flux mediated by the oxygen-binding proteins is constant at a particular protein concentration and does not depend on the oxygen partial pressure difference across the solution, thus this oxygen transport mechanism being dominant at low partial pressure provided that it remains sufficient to saturate the carrier. However, it was pointed out by Moll^12^ and Wyman^13^ that the facilitated oxygen transport can be effective if the kinetic time of the protein-oxygen binding reaction is significantly shorter than the diffusion time of the protein. The experimental proof of facilitated oxygen diffusion was evidenced by the increase of oxygen diffusion through a rather thick hemoglobin-water solution with respect of the one without Hb in solution, of the order of 100 *μm*. In this case, the diffusion time of the protein is much longer than the kinetic time of the oxygen-hemoglobin binding. The magnitude of facilitated diffusion and its relevance to gas transport was questioned by different authors, when the diffusion distance is reduced to a few 10 *μm* in cells, or even $\simeq$1 *μm* specifically in red blood cells (thickness of the cell).

The time the RBCs spend in the lungs near the alveolar sac, (i.e.: the transit or contact time) is usually assumed to span between 0.2 and 0.75 second depending on the activity of the subject (typically 0.7 sec at rest)^14, 15^. If we try to perform a mental experiment with the aim to maximize the oxygen transport by the RBCs, one should maximize the oxygen capture by the blood cells, which is performed during this limited time spent near the alveolus. Thus to optimize the transport of oxygen, different aspects must be taken into account. *i*-) With increasing the concentration of Hb in RBCs more oxygen can be transported (e.g. more binding sites for 0_2_ molecules). *ii*-) But as consequence of the high concentration, the diffusion of hemoglobin is strongly reduced and may become anomalous. One should also mention that, when Hb concentration increases, in addition to mobility reduction of the protein, the diffusion of oxygen is also reduced. Moreover, less volume is occupied by the “solvent” in the RBCs and for a given solubility of oxygen less oxygen is in present there, which also probably increases the oxygen time capture by the RBC, and consequently, decreases the amount of oxygen captured by hemoglobin during a limited contact time.

In this paper, we report a study of the nature of hemoglobin diffusion up to high concentration (Brownian or anomalous) by neutron spin-echo spectroscopy that shows that even at high protein concentration it remains of Brownian type. Provided this result, we address the question of the role of hemoglobin diffusion on oxygen capture by the red blood cells. Can a kinetic phenomenon counterbalance a mass effect in oxygen capture by the RBCs; is the time to catch oxygen by all the Hb molecules in RBCs significantly increased due to the reduced protein mobility? We use a model developed by Clark *et al*.^16^ where were computed the characteristic times for oxygen binding at the cellular level depending on different kinetic and diffusion times at a molecular level, to search for the optimum oxygen capture as a function of hemoglobin concentration. For this, we need a precise description of the variation of the diffusion mechanism of hemoglobin as a function of the concentration up to more than 330 *g*.*L* ^−1^ (physiological concentration), and to show that, at similar concentration of hemoglobin, the diffusion in concentrated solution and in the red blood cells are the same, to verify that hemoglobin diffusion in the RBC can be modeled by the one observed in solution.

Measurements of the self-diffusion coefficient of hemoglobin in solution have been reported in the literature, by tracer diffusion methods: diffusion through membrane^17–21^, by diffusion chamber method^17^, facilitated oxygen diffusion through hemoglobin solutions^22^, or by the measure of the oxygenation of a deoxygenated layer of hemoglobin by photometric method after a sudden change of oxygen partial pressure^23^. It has also been measured by pulse-field Gradient Nuclear Magnetic resonance (PFG-NMR)^24, 25^ but the question of a possible anomalous behavior was not addressed. The diffusion of hemoglobin in red blood cells was measured by PFG-NMR^26, 27^ and more recently by neutron spin echo spectroscopy^28^ (NSE). It was stressed by Bouwer *et al*.^22^, by a common plot of several experimental results, that there is a strong scattering of the data, especially at high protein concentration, with more than a factor of 10 sometimes between the diffusion coefficients given for hemoglobin at similar concentration. It was previously shown ref. 24 that the temperature variation of the diffusion coefficient in protein solution scales well with the viscosity of water, following the Stokes-Einstein equation. It is thus possible, by a simple scaling with the viscosity in *H*_2_ *O* and *D*_2_ *O*, to compare the different experiments. In both NMR experiments in RBCs the authors used *D*_2_ *O* suspensions. In ref. 27 the measurements were performed at *T* = 25 °C. After correction for temperature dependence of the *D*_2_ *O* viscosity and assuming Stokes-Einstein like behavior of the different parameters, we estimate ${D}_{s}\simeq 0.93\pm 0.16\,{10}^{-7}$ *cm* ^2^.*s* ^−1^ at 37 °C in *H*_2_ *O*. In the older measurement of ref. 26 a value of ${D}_{s}\simeq 2.2\pm 0.1\,{10}^{-7}$ *cm* ^2^.*s* ^−1^ can be calculated at 37 °C in *H*_2_ *O* using similar procedure. These results are at odds and our measurement of *D*_*s*_ of hemoglobin in RBCs, obtained by Neutron Spin-Echo (NSE), lie just in between^28^ with *D*_*s*_ = 1.75 10^−7^ *cm* ^2^.*s* ^−1^. Notice that in ref. 27 a concentration of hemoglobin of 355 *g*.*L* ^−1^ in the RBCs was given, whereas in ref. 26 nothing is said, we thus assumed a concentration corresponding to the average observed for human of 330 *g*.*L* ^−1^. Back in 1966, Moll^17^, by milipor filter membrane method and diffusion chamber method, estimated the diffusion coefficient of hemoglobin solutions at physiological concentration (not in the RBCs) to *D*_*s*_ = 0.6410^−7^ and to *D*_*s*_ = 0.45 10^−7^ *cm* ^2^.*s* ^−1^, showing significant differences between the diffusion coefficients in the RBCs and at similar concentrated water-based solutions. However, our results on the diffusion coefficient measured by NSE in the RBCs is very close to the one given by Bouwer *et al*.^22^ which can be calculated to *D*_*s*_ = 1.5 − 1.6 10^−7^ *cm* ^2^.*s* ^−1^ at *T* = 37 °C. In order to get a precise evolution of the diffusion of hemoglobin as a function of the concentration we performed a Neutron Spin Echo study of the hemoglobin diffusion in concentrated solutions ranging from a few tens of *g*.*L* ^−1^ up to physiological concentration. By NSE, we measure the Intermediate Scattering Function (ISF) and thus we are able to address the question of the Brownian or anomalous nature of the diffusion mechanism.

## Results and Analysis

### Brownian versus anomalous diffusion

By NSE we measure the intermediate scattering function *I*(*q*, *t*). For coherent scattering (as is the case for protein solutions in *D*_2_ *O*) it corresponds to a pair-correlation function:

1 $$I(q,t)=\sum _{i,j}\,\langle {b}_{i}{b}_{j}{e}^{-iq\mathrm{.(}{r}_{j}(t)-{r}_{i}\mathrm{(0))}}\rangle $$

q is the scattering wave vector, with *q* = (4*π*/*λ*) *sin* (*θ*), where 2*θ* is the scattering angle and *λ* the wavelength of the neutrons, *r*_*j*_(*t*) is the position of the jth *scattering center* at a time t. *b*_*i*_ is the coherent scattering length of the i-th scattering center.

For almost spherical macromolecules, like hemoglobin, at *very low concentration*, experiencing Brownian motion in the solvent, the intermediate scattering function, in the Small-Angle Neutron Scattering (SANS) regime ($q\ll \tfrac{2\pi }{d}$, d is the distance between two scattering centers) can be reduced to a self-correlation function^29, 30^:

2 $${I}_{s}(q,t)\simeq \sum _{i,j}\,\langle {e}^{-iq\mathrm{.(}{r}_{i}(t)-{r}_{i}\mathrm{(0))}}\rangle \simeq {e}^{-\frac{{q}^{2}\langle {r}^{2}(t)\rangle }{6}}$$

*r*_*i*_(*t*) is now the position of the ith *centre of mass* of the protein. In this particular case, the rotational Brownian motion can be neglected. The ISF is sensitive to only the translational Brownian motion of the protein with $\langle {r}^{2}(t)\rangle =6{D}_{s}t$ and thus

3 $${I}_{s}(q,t)\simeq {e}^{-{D}_{s}{q}^{2}t}$$

where *D*_*s*_ is the self-diffusion coefficient defined in Stokes-Einstein equation (see ref. 31 for details). For Brownian motion in the SANS domain, *I*(*q*, *t*) is a single exponential decay, ${I}_{s}(q,t)={e}^{-\frac{t}{\tau }}$ with $\frac{1}{\tau }={D}_{s}{q}^{2}$.

When increasing the concentration of macromolecules, interaction between them must be taken into account and the *i* ≠ *j* terms in equation 1 cannot be neglected, a protein-protein structure factor different than 1 appears in the solution scattering intensity. However in the wavevector range $q.R\gg 1$, where R is radius of the protein, one gets *S*(*q*) ~1, the *i* ≠ *j* terms in equation 1 can be neglected; in this wavevector range one measure the self-intermediate scattering function *I*_*s*_(*q*, *t*) of a concentrated protein solution. In addition to this interferences effects, the diffusion may become anomalous, $\langle {r}^{2}(t)\rangle =6{D}_{s}{t}^{\alpha }$ where *α* > 1 corresponds to superdiffusion and *α* < 1 to subdiffusion, which is generally expected in cells. *α* < 1 leads to a stretching in the self-intermediate scattering function ${I}_{s}(q,t)\simeq {e}^{-{q}^{2}{D}_{s}{t}^{\alpha }}$ and thus for anomalous diffusion, the spectra are refined with a usual stretched exponential function: ${I}_{s}(q,t)={e}^{-{(\tfrac{t}{\tau })}^{\alpha }}$ and one obtain a characteristic wavevector dependence of the inverse relaxation time $\frac{1}{\tau }\sim {q}^{\frac{2}{\alpha }}$ ^30, 32^.

Thus, from the study of the intermediate scattering in the appropriate wavevector regime ($q\mathrm{.}R\gg 1$), one can clearly distinguish between Brownian and anomalous diffusion^30, 32, 33^.

### Experimental results and analysis

The spectra were first refined using a stretched exponential function:

4 $$I(q,t)=A(q){e}^{-{(\tfrac{t}{\tau })}^{\beta }}$$

in order to distinguish between a single exponential decay or a stretched exponential of the intermediate scattering function (we use *β* rather than *α* as is generally found in the literature^32^, but it has the same significance). We clearly verified that $\beta \simeq 1$ for the range of wavevector of interest (see SI), which highlights the single exponential decay of *I*(*q*, *t*). Notice that at the smallest wavevectors *β* tends to decrease slowly, but in this q range we do not measure a self-correlation function but a pair correlation. For a single exponential decay, we usually introduce a wavevector dependent diffusion coefficient *D*(*q*) which accounts for the change of the diffusion process depending on the distances:

5 $$I(q,t)={e}^{-D(q){q}^{2}t}$$

**Figure 1:**
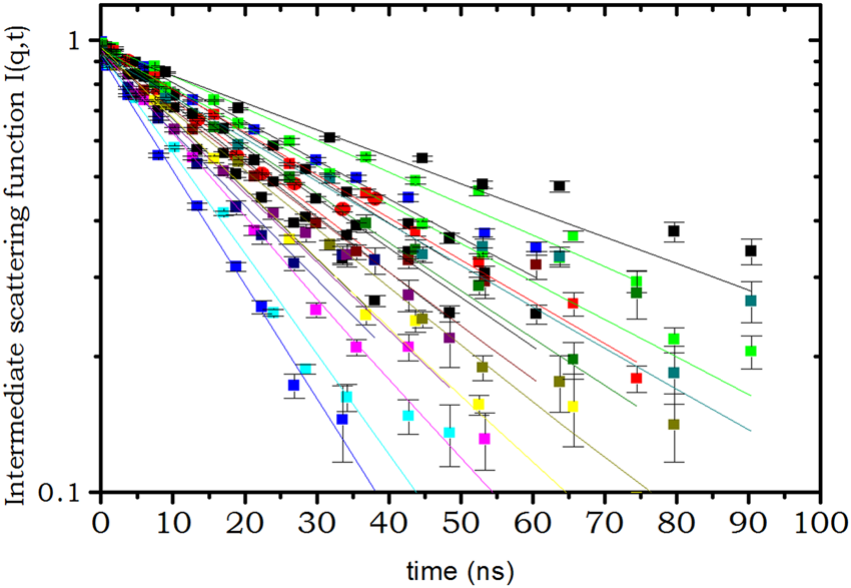
Intermediate scattering function measured on the SNS neutron spin echo spectrometer, at wavevector ranging from q = 0.069 *Å* ^−1^ to q = 0.125 *Å* ^−1^, on hemoglobin solution of *c*_*p*_ = 105 *g*.*L* ^−1^. 22 spectra are shown from the 60 that has been measured on 4 different scattering angles.

**Figure 2:**
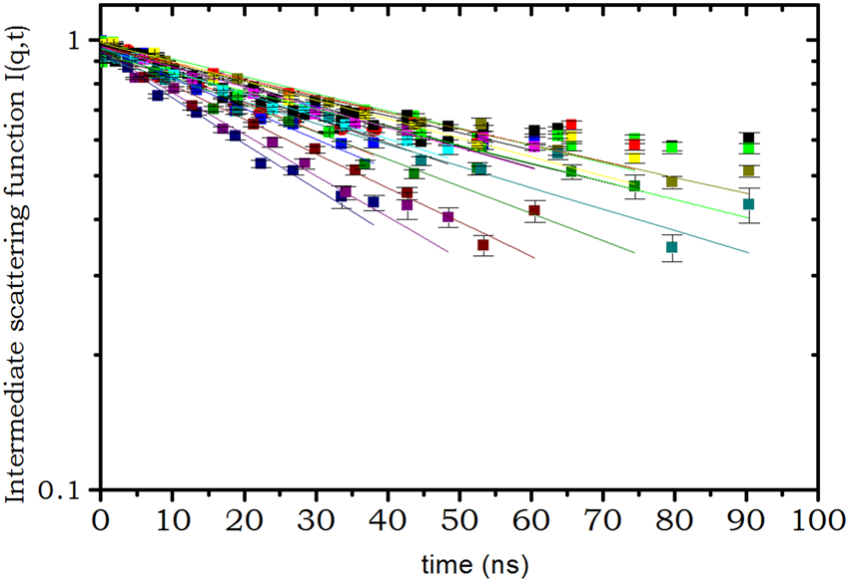
Intermediate scattering function measured on the SNS neutron spin echo spectrometer, at wavevector ranging from q = 0.069 *Å* ^−1^ to q = 0.25 *Å* ^−1^, on hemoglobin solution of *c*_*p*_ = 327 *g*.*L* ^−1^.

**Figure 3:**
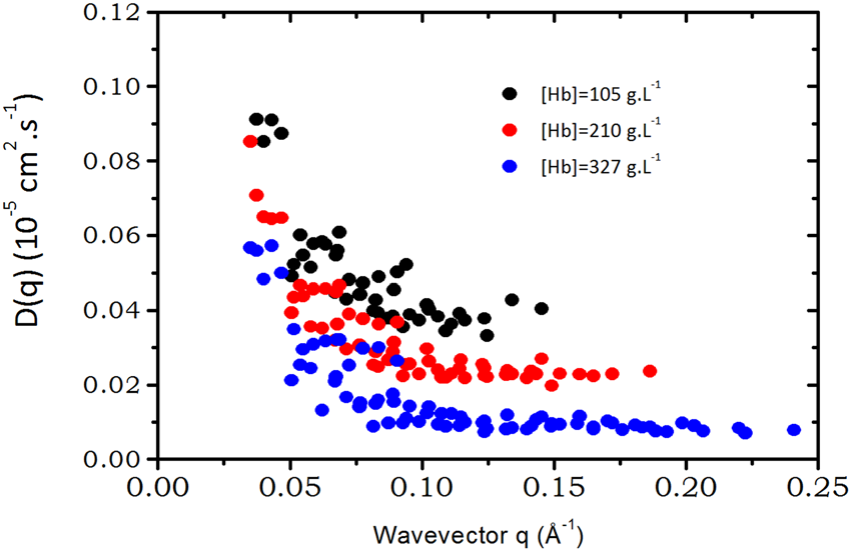
Wavevector dependence of the apparent diffusion coefficient D(q) measured by NSE on three solutions of concentration *c*_*p*_ = 105 *g*.*L* ^−1^, *c*_*p*_ = 210 *g*.*L* ^−1^ and *c*_*p*_ = 330 *g*.*L* ^−1^. The value obtained for *q* → 0 is the mutual or collective diffusion coefficient whereas the value of the plateau corresponds to the long time self-diffusion coefficient at a given concentration *D*_*s*_(*c*_*p*_) (see text).

*D*(*q*) is called the apparent diffusion coefficient. So, in a second series of refinements we fitted formula 5 to the experimental data and extracted *D*(*q*, *c*_*p*_) for each solution of hemoglobin at different concentration. The intermediate scattering function measured on hemoglobin solutions by the neutron spin-echo together with the fits are depicted on Figs 1 and 2 for two protein concentrations *c*_*p*_ = 105 *g*.*L* ^−1^ and *c*_*p*_ = 327 *g*.*L* ^−1^. For clarity only a limited number of spectra are plotted over all measured. When increasing the concentration, due to the decrease of the diffusion (ie. increase of the relaxation times) we had to increase the range of wavevectors measured to get a faster decrease of *I*(*q*, *t*). For both figures it is clear that there is a slight departure from the single exponential decay for the smallest q measured (longest relaxation times). The results obtained for *D*(*q*, *c*_*p*_) are depicted on Fig. 3 for three concentrations: *c*_*p*_ = 105 *g*.*L* ^−1^, *c*_*p*_ = 210 *g*.*L* ^−1^ and *c*_*p*_ = 327 *g*.*L* ^−1^.

Since coherent neutron scattering is measured, at very small wave vector, *D*(*q*, *c*_*p*_) is a collective diffusion coefficient, in the limit of *q* → 0 thus similar to the one measured by Photon Correlation Spectroscopy (PCS). This collective diffusion coefficient was measured in hemoglobin solutions, and vary very slowly with concentration^34, 35^. Unfortunately, it is not possible to compare PCS measurements of Hb diffusion in the RBCs with NSE results because light scattering spectra are dominated by membrane fluctuations^36^. The collective diffusion coefficient is significantly larger than the self-diffusion one^35^, especially at high protein concentration, and both collective and self-diffusion coefficients match at infinite dilution.

**Figure 4:**
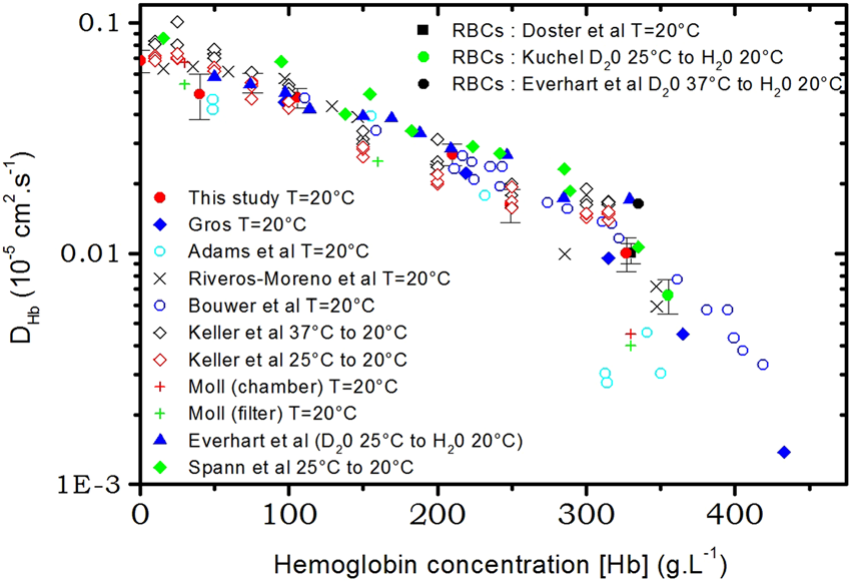
Experimental results of the variation of the self-diffusion coefficient in solution obtained by different methods, big full red circle this study, from Moll^17^ green and red crosses^17^, Gross^21^ blue diamond, Riveros *et al*.^20^ black cross, Everhart and Johnson^25^ blue triangle, Keller *et al*. *T*_*c*_ = 37 °C to *T*_*c*_ = 20 °C empty black diamond, *T*_*c*_ = 20 °C empty red diamond, Spaan *et al*.^23^ full green diamond, Adams and Fatt^18^ full cyan circle and Bouwer *et al*.^22^ empty blue circle. And self-diffusion coefficient measured directly in the RBCs by NMR full black circle^26^, full green circle^27^ and NSE in the RBCs full black square^28^.

When *D*(*q*) is a constant as a function of the wavevector q, it means that the relaxation time extracted from the intermediate scattering function has a *q* ^2^ dependence; $I(q,t)={e}^{-\frac{t}{\tau }}$ with $\frac{1}{\tau }={D}_{s}{q}^{2}$ (see formula 5). The value obtained at high q (value of the plateau) corresponds to the wavevector range where the protein-protein structure factor is equal to $S(q)\simeq 1$. As was mentioned before, this q range corresponds to the incoherent approximation of coherent scattering, and the apparent diffusion coefficient corresponds to the self-diffusion ${D}_{s}({c}_{p})={\mathrm{lim}}_{q\mathrm{.}R\gg 1}\,D(q,{c}_{p})$. It was shown previously that it corresponds to the long time self-diffusion coefficient^28, 37^. The variation of the self-diffusion coefficient for hemoglobin solutions at different concentration measured by neutron spin-echo at *T* = 20 °C is depicted on Fig. 4. For comparison, experimental results obtained by different techniques are plotted together after rescaling to *T* = 20 °C following Stokes-Einstein relation (see above). It is striking that there is a strong scattering of the points obtained by different authors and different experimental techniques.

In the case of protein in the the RBCs and in solutions where the concentration of hemoglobin is almost constant apart small fluctuations, the long-time self-diffusion coefficient must be used to evaluate transport properties. The strong deformation of the RBCs would probably lead the collective protein diffusion more relevant. However, we decided to neglect this effect and assume that the transport diffusion coefficient in RBCs corresponds to the self-diffusion one, which is accessible by NSE at higher wave vectors^38, 39^.

### Analytical description of the concentration dependence of the diffusion coefficient

The viscosity of concentrated protein solution are generally described using the equation derived by Mooney^40^ which is an extension to finite concentration of the Einstein formula for infinite dilution solution of rigid spherical particles:

6 $$\eta ={\eta }_{0}\,exp\,(\frac{\nu {\rm{\Phi }}}{1-k{\rm{\Phi }}})$$

*η*_0_ is the viscosity of the solvent, Φ is the volume fraction of the protein and k is a constant, the self-crowding factor. The factor *ν* is defined by $\nu \eta ={\mathrm{lim}}_{{\rm{\Phi }}\to 0}\,\frac{\eta -{\eta }_{0}}{{\eta }_{0}}$, it is a generalization Einstein equation $\eta \simeq {\eta }_{0}\mathrm{(1}+2.5\varphi )$, it was set to *ν* = 2.5 by Mooney to match Einstein formula, but can exceed this value for non-spherical particles. A difficulty for the application of this formula to protein solutions is to evaluate Φ, because it corresponds to an hydrodynamic volume fraction that includes the shell of water of hydration which moves with the protein core, that is hardly defined. Ross and Minton^41^ overcome this difficulty by modifying the Mooney equation. They introduced the intrinsic viscosity of the solution [*η*], a quantity measured from a dilute solution of macromolecule that contains information on the macromolecular shape, which is defined as:

7 $$[\eta ]=\mathop{\mathrm{lim}}\limits_{c\to 0}\frac{\eta -{\eta }_{0}}{c{\eta }_{0}}$$

[*η*] is familiar to molecular biophysicist and can be measured for protein solutions^42^. Finally, Ross and Minton derived a modified Mooney formula where the volume fraction of the protein has been replaced by its concentration:

8 $$\eta ={\eta }_{0}\,exp\,(\frac{[\eta ]{c}_{p}}{1-(k/\nu ){c}_{p}[\eta ]})$$

**Figure 5:**
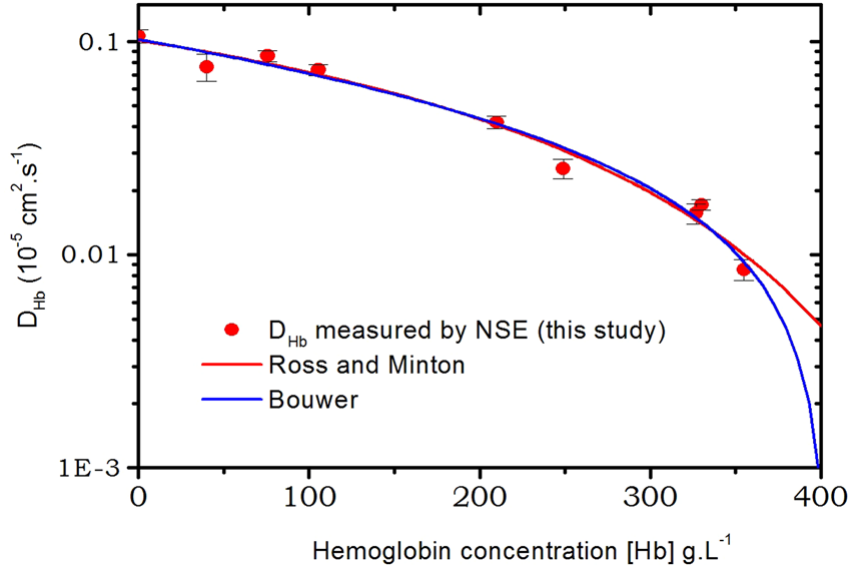
Self-diffusion coefficient *D*_*s*_(*c*_*p*_) measured by NSE spectroscopy (red circles) and refinements using the modified Mooney formula given by Ross and Minton^41^ (red line) and empirical formula used by Bouwer *et al*.^22^ (blue line) (see text).

The Fig. 5 depicted the fits by a formula similar to 8, (*D* ~ 1/*η*) of the results of the diffusion coefficient obtained from NSE spectroscopy during this measurement, we also plotted the results obtained in RBC by the same method^28^ and by NMR^27^. Notice that by assuming *D*_*s*_(*c*_*p*_) · *η*(*c*_*p*_) = *cste* we assume the validity of the generalized Stokes-Einstein equation as is usually assumed of verified in crowded protein solutions^43^ (for a critical discussion on the validity of the generalized Stokes-Einstein relation see ref. 44). All the results has been corrected for the viscosity of *D*_2_0 to *H*_2_0 and scaled to a temperature of *T*_*c*_ = 37 °C using the procedure described previously. The values extracted from the fitting procedure are the infinite dilution hemoglobin diffusion coefficient at *T* = 37 °C: *D*_*s*_(0) = 10.1 ± 0.5 10^−7^ *cm* ^2^.*s* ^−1^, the intrinsic viscosity [*η*] = 2.94 ± 0.79 10^−3^ *L*.*g* ^−1^ and the ratio *k*/*ν* = 0.52 ± 0.29.

We also refined the data using the empirical equation used by Bouwer *et al*.^22^:

9 $$D({c}_{p})={D}_{s}\mathrm{(0)}\,(1-\frac{{c}_{p}}{{c}_{1}}{10}^{-\frac{{c}_{p}}{{c}_{2}}})$$

The results of the refinements are also plotted on Fig. 5, we obtained for the diffusion coefficient at zero concentration *D*_*s*_(0) = 10.2 ± 0.6 10^−7^ *cm* ^2^.*s* ^−1^, and for the concentration *c*_1_ and *c*_2_ respectively *c*_1_ = 404 ± 43 *g*.*L* ^−1^ and *c*_2_ = 2684 ± 3188 *g*.*L* ^−1^. Both Ross and Minton equation and Bouwer empirical formula give very similar variation of the self-diffusion coefficient of hemoglobin up to ${c}_{p}\simeq 350\,g.{L}^{-1}$ and only differ significantly above. They both match relatively accurately with the experimental results.

## Discussion

### Brownian versus anomalous diffusion of hemoglobin

Since *I*(*q*, *t*) follow a single exponential decay with $\frac{1}{\tau }={D}_{s}{q}^{2}$ in the wavevector domain where the self-correlation function is measured, we can clearly conclude that whatever the concentration that is measured, the diffusion of hemoglobin remains of Brownian type, although with a strong reduction of the diffusion coefficient. This result can be compared with the measurements reported in ref. 6 where it is observed that in the cytoplasm below a diffusion distance of 100 nm the diffusion of GFP (green fluorescent protein) in of Brownian type where as it becomes anomalous above. This characteristic distance in the change of diffusion behavior was attributed to the presence of immobile structure in the cytoplasm. In our measurements, the diffusion processes are probed over much smaller distances (typically a few nm) and due to the specificity of the RBCs (very homogeneous cell filled with one almost spherical protein) the transition from Brownian to anomalous diffusion, that is attributed to structural inhomogeneity of the cytoplasm, may be absent. In homogeneous colloidal suspensions^37^ and protein solution^7^ a transition from Brownian to anomalous diffusion is observed at high volume fraction (Φ ~ 0.5–0.6), which is well above the volume fraction of hemoglobin in the RBCs or in the solutions measured in the present study: ${\rm{\Phi }}\simeq 0.25$ for dry protein physiological concentration and ${\rm{\Phi }}\simeq 0.36$ when including the protein hydration shell with h = 0.35 gram of water per gram of protein^45^. It was suggested from experimental results^28^ and supported by numerical simulation^46^ that the strong reduction of the diffusion coefficient when increasing protein concentration can mainly be attributed to hydrodynamic interactions rather than direct interactions.

### Concentration dependence of the diffusion coefficient of hemoglobin

The variation of the diffusion coefficient found in the literature, as is depicted on Fig. 4, shows very strong dispersion especially at high concentrations, therefore we decided to measure it by Neutron Spin Echo, although we are aware that it is not the easiest way to do it. But due to the very well defined neutron-matter interaction, we have a very precise direct measurement of *D*_*s*_(*c*_*p*_) provided the fact that we measure at sufficiently high wavevector in order to extract the self-diffusion coefficient. Of course, this is true if the quality of the data (mainly due scattered neutron flux and beam polarization) allows a good measurement of intermediate scattering function. Up to concentration in protein up to *c*_*p*_ = 330 *g*.*L* ^−1^ which corresponds to physiological concentration our results are basically situated around the average values of the literature very close to the more recent ones measured by Bouwer *et al*.^22^. But very importantly, we show that the measurements performed in RBCs and in solution at the same concentration are the same. Equations 8 and 9, used to describe the variation of the diffusion coefficient as a function of the concentration give very similar results up to concentrations of 350 *g*.*L* ^−1^. They significantly differ at higher concentrations but it is difficult to achieve experimentally because highly concentrated samples are difficult to obtain due to increase viscosity of the solution.

### Influence of the diffusion on the oxygen capture of RBCs

The relevance to biological activity of hemoglobin diffusion as a function of the concentration and inside erythrocytes concerns the kinetics of oxygen uptake and release of the RBCs. A discussion on typical time scales for oxygen uptake and release is given in ref. 16. Following the authors, the internal time scale *τ*_1_ for the capture (or release) of oxygen within the red cell can be estimated by:

10 $${\tau }_{1}=\frac{{\tau }_{KC}+{\tau }_{KS}}{{\tau }_{KS}/{\tau }_{KC}+{\tau }_{KC}/{\tau }_{DC}}$$

*τ*_*KS*_ = *k* ^−1^ is the dissociation rate constant of the chemical reaction $Hb+{O}_{2}\rightleftarrows Hb{O}_{2}$ with k = 44 *s* ^−1^.

*τ*_*KC*_ = (*kN*_*T*_/*N*_50_)^−1^ corresponds to the time necessary for half of the hemoglobin content to catch oxygen at a given oxygen partial pressure in the plasma. *N*_*T*_ is the hemoglobin density, and *N*_50_ the *O*_2_ density necessary to have 50% of hemoglobin to be bonded.

${\tau }_{DS}=\tfrac{{a}^{2}}{{D}_{Hb}}$ is the characteristic time scale for hemoglobin to diffuse from the cell center to the surface or vice versa (depending on uptake or release). The RBC shape is assumed to be disks of radius R, and height 2a with 2a $\ll$ R). *D*_*Hb*_ is the diffusion coefficient of hemoglobin.

${\tau }_{DC}=\tfrac{{a}^{2}}{{D}_{o}}$ is the characteristic time scale for oxygen to diffuse from to the cell center to the surface. ${D}_{{O}_{2}}$ is the self-diffusion coefficient of molecular oxygen.

From this, calculations can be done to address the question: what is the optimum hemoglobin concentration in RBCs to transport a maximum of oxygen? As was mentioned before, the time a RBC spends in the lungs near the alveolar sac is usually assumed to span between 0.2 and 0.75 second. The more hemoglobin is in RBCs the more oxygen can be transported. But as consequence, the diffusion coefficient of hemoglobin and oxygen are strongly reduced.

Three parameters depend on hemoglobin concentration (*c*_*p*_): *N*_50_(*c*_*p*_), *D*_*Hb*_(*c*_*p*_) and ${D}_{{O}_{2}}({c}_{p})$ (we use *c*_*p*_ as the hemoglobin concentration in *g*.*L* ^−1^). The first Hb concentration dependent parameter, *N*_50_(*c*_*p*_) (the *O*_2_ density necessary so that 50% of hemoglobin to be bonded at a given *c*_*p*_), is given by ref. 16. The kinetics of oxygen binding by hemoglobin is described by the Hill equation:

11 $$S=\frac{{(P/{P}_{50})}^{n}}{1+{(P/{P}_{50})}^{n}}$$

with *P*_50_ = 26.4 mmHg, n = 2.65. Its hemoglobin volume fraction dependence is given in ref. 16:

12 $${N}_{50}({c}_{p})=3.72\ast {10}^{-8}\mathrm{(1}+0.000312{c}_{p})$$

We used concentration analytical function to describe the dependence of the hemoglobin diffusion coefficient *D*_*Hb*_(*c*_*p*_) described before using the modified Mooney formula^41^ computed above from *I*(*q*, *t*) analysis.

To a less extent the diffusion coefficient of molecular oxygen depends on the Hb concentration too. Clark *et al*.^16^ used a value at 37 °C in RBCs of ${D}_{{O}_{2}}^{RBC}=9.5\ast {10}^{-6}$ *cm* ^2^.*s* ^−1^ this was estimated from the data summarized by Himmelblau^47^, whereas ${D}_{{O}_{2}}=2.73\ast {10}^{-5}$ *cm* ^2^.*s* ^−1^ in pure *H*_2_ *O* at physiological temperature. The dependence of the oxygen diffusion coefficient was measured at 20 °C in different hemoglobin solutions by Bouwer and coworkers^22^. Finally after usual temperature corrections, for a temperature of T = 37 °C, we took a hemoglobin concentration dependence of the oxygen diffusion coefficient to be:

13 $${D}_{{O}_{2}}({c}_{p})={D}_{{O}_{2}}(1-\frac{{c}_{p}}{{C}_{1}}){10}^{-{c}_{p}/{C}_{2}}$$

with ${D}_{{O}_{2}}=2.73\ast {10}^{-5}$ *cm* ^2^.*s* ^−1^, *C*_1_ = 100 *g*.*L* ^−1^ and *C*_2_ = 119 *g*.*L* ^−1^ given by ref. 22.

The characteristic time *τ*_1_ for oxygen capture can then be easily computed and, knowing that the RBC have a limited time of *τ*_*r*_ in the lung capillaries, we can roughly estimate the number density of Hb which load oxygen as a function of the Hb concentration by:

14 $$\tilde{N}({c}_{p})=N({c}_{p})\ast exp(-{\tau }_{1}/{\tau }_{r})$$

**Figure 6:**
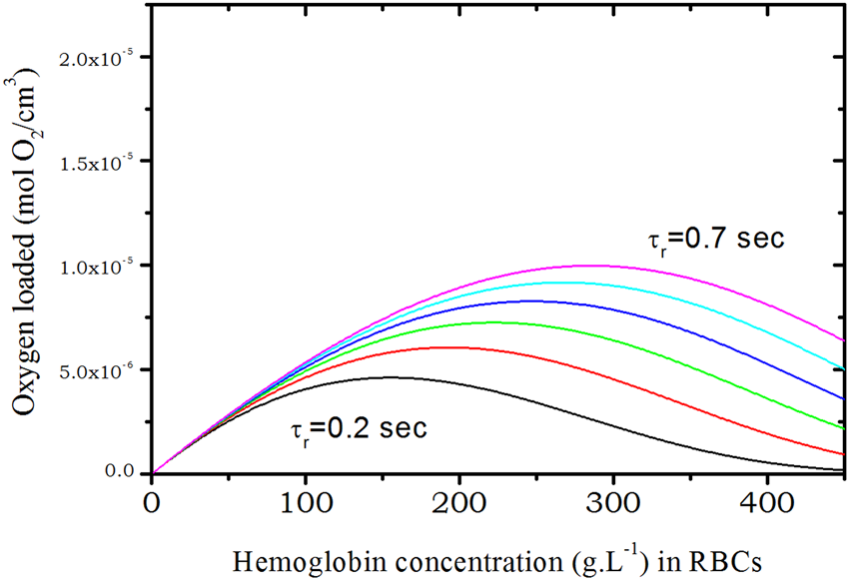
Number of moles of oxygen loaded by hemoglobin per *cm* ^3^ in the red blood cells as a function of the hemoglobin concentration in *g*.*cm* ^−3^ for different transit times. We assume that at time t = 0 there was no *O*_2_ bounded and that the oxygenation is performed within a transit time varying from 0.2 to 0.7 sec without Hb diffusion.

**Figure 7:**
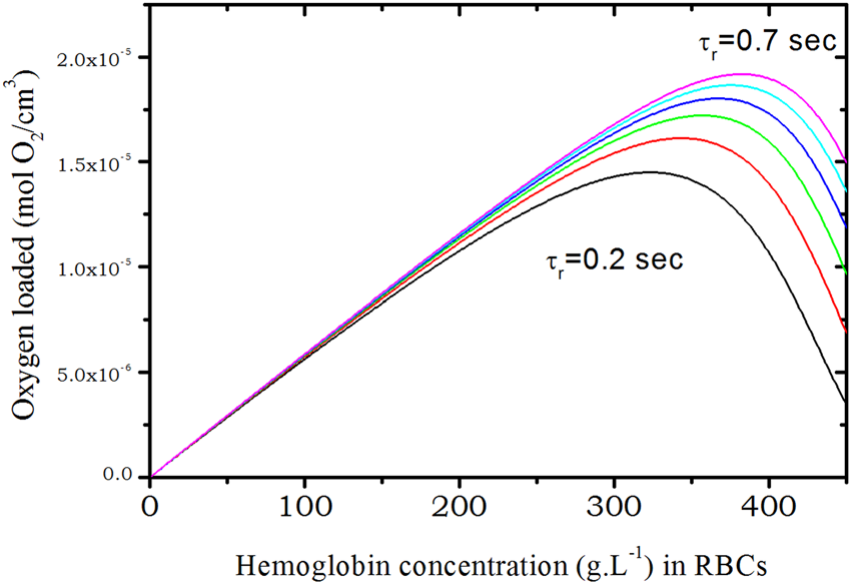
Same as Fig. 6 but this time computed with including the concentration dependence of Hb diffusion coefficient.

*N*(*c*_*p*_) is the number of heme (*O*_2_ capture site) in one RBC, *τ*_*I*_ and *τ*_*r*_ are respectively the internal and residence times scale, $\tilde{N}({c}_{p})$ is the number of *O*_2_ loaded in the RBC. We are, of-course, aware that to describing oxygen capture as a single exponential process is a rather crude approximation but this has to be regarded as a first order approximation. $\tilde{N}({c}_{p})$ is depicted on Fig. 6 assuming that Hb molecules have fixed positions in space (*D*_*Hb*_ = 0), and using the measured concentration dependence of the diffusion coefficient on Fig. 7. From this very simple calculation we first can state that Hb diffusion inside erythrocytes clearly helps in increasing *O*_2_ transports since there is a factor of 2 to 3 between the maxima of the curves for the same residence time. The second and more striking fact is that the optimum hemoglobin concentration in RBCs with respect to maximizing oxygen transport is very close to the physiological one for an individual under strong physical activity, whereas without including the diffusion of hemoglobin, at physiological Hb concentration, the optimum is observed for an individual at rest, that is for a residence time of *τ*_*r*_ = 0.7–0.8 sec.

The biconcave shape of the RBCs is usually explained by two approaches. First, the molecular view, which assumes that this shape is driven by the molecular structure of the membrane, and second, the mechanistic view. The second approach states that torsion of the cells having biconcave shape is only low energy consuming and hence favor a decrease of blood viscosity especially in the capillaries whose diameters are close to or smaller than the red cells dimensions. For oxygen capture the characteristic length scale is *a*, which represents in the model the half average distance between the two membrane surfaces of the biconcave characteristic RBC shape. We can speculate that the biconcave shape could also serve to minimize the distance between hemoglobin and the cell surface and consequently reduce the oxygen capture time, although the biconcave shape is not observed in all mammalians.

## Conclusion

In summary, we have performed a complete Neutron Spin Echo study of the concentration dependence of the diffusion of hemoglobin in solution and verified that *i*- the diffusion remains of Brownian type whatever the concentration of the protein in solution, *ii*- we could extract the concentration dependence of the diffusion coefficient of hemoglobin *D*_*s*_(*c*_*p*_) up to physiological concentration and *iii*- we verified that the diffusion coefficient are the same in the RBCs and in solution at similar concentration. The variation of *D*_*s*_(*c*_*p*_) can be fitted to the analytical formula 8 and 9 up to physiological concentration found in the RBCs. We have also shown that with a simple calculation using the characteristic time *τ*_1_ for oxygen capture (or release) given by Clark *et al*.^16^, and including the measured concentration dependence of the 3 parameters *N*_50_(*c*_*p*_), *D*_*Hb*_(*c*_*p*_) and ${D}_{{O}_{2}}({c}_{p})$, it is possible to show that *i*- the diffusion of hemoglobin facilitate the oxygen capture of the RBCs, and that *ii*- the hemoglobin concentration in the RBCs corresponds to an optimum of oxygen capture for an individual under physical activity.

## Methods

### Sample preparation

Human hemoglobin was purchased from Sigma-Aldrich (H73–89). It was dissolved in *H*_2_ *O* at roughly 10 *g*.*L* ^−1^ and then concentrated by centrifugation up to 100 *g*.*L* ^−1^. Different solutions of volume 15 ml were then dialized against 300 ml of *D*_2_ *O* (for contrast reason in neutron scattering). This operation was repeated 3 times for each solution to remove the labil protons exchanged from the protein in the solvent. The solutions were then concentrated to that measured by neutron spin echo spectroscopy: 40, 75, 105, 210, 250 and 330 *g*.*L* ^−1^. The estimated concentrations were verified by thermogravimetric analysis (TGA Q50 from TA instruments).

### Neutron Spin Echo

By Neutron Spin Echo spectroscopy^48^ one measure the intermediate scattering function of the sample *I*(*q*, *t*). A polarized neutron beam is submitted to a suit of magnetic fields (of similar integrals) before and after the sample. If the scattering process is elastic (no energy exchange between the sample and the neutron) the beam polarization should remain constant as a function of the magnetic field intensity (apart from intrinsic depolarization due to the spectrometer inhomogeneity). If the scattering is inelastic with a small energy transfer distribution the beam (the energy exchanged between the neutron and the sample is much smaller than the neutron one) the scattered beam polarization will decrease in a similar way to the intermediate scattering function. A detail of experimental setup can be found in different text books^49, 50^. The Neutron Spin Echo experiments were performed at the Spallation Neutron Source at Oak-Ridge, Tennessee (USA), on the Juelich center for Neutron Scattering (JCNS) spin-echo spectrometer^51^. This spectrometer, due to its situation at a pulsed source, works in the time-of-flight mode (TOF)^52^. The advantage of this method is that it allows simultaneously to measure over different wavelength *λ*, and consequently over different time ranges since $\tau \sim {\rm{\Phi }}{\lambda }^{3}$, where Φ is the field path integral in the coils. The wavelength band covered for the experiments was spanning from *λ*_*min*_ = 6.94 *Å* to *λ*_*max*_ = 9.96 *Å*, the sample detector distance was set to *L*_*SD*_ = 3.94 m. The neutron are counted on a 32 ∗ 32 detector over 42 time channels. Each echo is measured over 27 points (19 points per echo, 3 for down and 5 for up limits of the echo amplitude) and 10 echos are measured per intermediate scattering function, corresponding to ten different sets of currents. The data treatment was performed with in-house developed procedure using Matlab@. The detector is grouped into 3 Debye-Sherrer rings and the TOF channels are reduced from 42 to 5 (by grouping). Thus 15 intermediate scattering function are obtained per configuration (one measured scattering angle 2*θ*). Each of the five TOF channels corresponds to an average wavelength and the Fourier times are computed from the field path integrals. The highest wavectors (shortest wavelengths) cover a time range from *τ* = 0.08 ns to *τ* = 38.14 ns whereas for the smallest wavectors (highest wavelengths) the times span from *τ* = 0.20 ns to *τ* = 90.48 ns. In order to get a significant signal from the macromolecules, the protein are dissolved in *D*_2_0 and coherent neutron scattering is measured in the small angle regime.

The solutions were tested by small angle neutron scattering (SANS) in order to verify the lack of protein aggregation and the low level of hydrogen remaining in solution.

## Electronic supplementary material

Supporting information
