# Supplementary material for: Hemoglobin diffusion and the dynamics of oxygen capture by red blood cells
Source: Sci Rep. 2017 Sep 5;7:10448. doi: 10.1038/s41598-017-09146-9 (PMC5585185; doi:10.1038/s41598-017-09146-9)
Supplement: Supplementary file 1 — Supporting information [file 41598_2017_9146_MOESM1_ESM.pdf]

Hemoglobin diffusion and the dynamics of oxygen capture by red blood cells †

Stéphane Longeville*,‡ and Laura-Roxana Stingaciu¶

‡*Laboratoire Léon-Brillouin, CEA, CNRS, Université Paris-Saclay, CEA Saclay, F-91191 Gif-sur-Yvette, France*

¶*Juelich Centre for Neutron Science, outstation at SNS, Oak Ridge National Laboratory, Oak Ridge, TN 37831, USA.*

E-mail: slongeville@cea.fr

Phone: +33 (0)1 69 08 75 30. Fax: +33 (0)1 69 08 82 61

Supporting Information (SI)

Small Angle Neutron scattering

The Small Angle Neutron Scattering experiments were carried out on the spectrometer PACE of the Laboratoire Léon Brillouin in France. We used a wavelength of 6 Å given by a velocity selector with a wavelength band of $\delta\lambda/\lambda \simeq 0.1$. Two configurations were used order to cover the full wavevector range relevant for the experiment, with sample to detector distances of $L_{SD} = 1$ m and $L_{SD} = 3$ m. The wavevector ranges were respectively of $q = 0.02 - 0.21$ Å^{-1} and $q = 0.05 - 0.47$ Å^{-1}. For a solution of almost spherical macromolecules the scattering intensity can be written as:[1]

†Supporting informations

$$I(q) = v_p \Phi (\Delta\rho)^2 F^2(q) S(q) \tag{1}$$

v_p is the volume of the macromolecule, Φ is the volume fraction of the macromolecule in solution , $\Delta\rho$ is the scattering length density contrast between the macromolecule and the solution in cm^{-2}. $F(q)$ is the form factor of the macromolecule and $S(q)$ is the protein-protein structure factor of the solution.

SI Figures

References

(1) Chen, S. H. *Ann. Rev. Phys. Chem.* **1986**, *37*, 351–399.

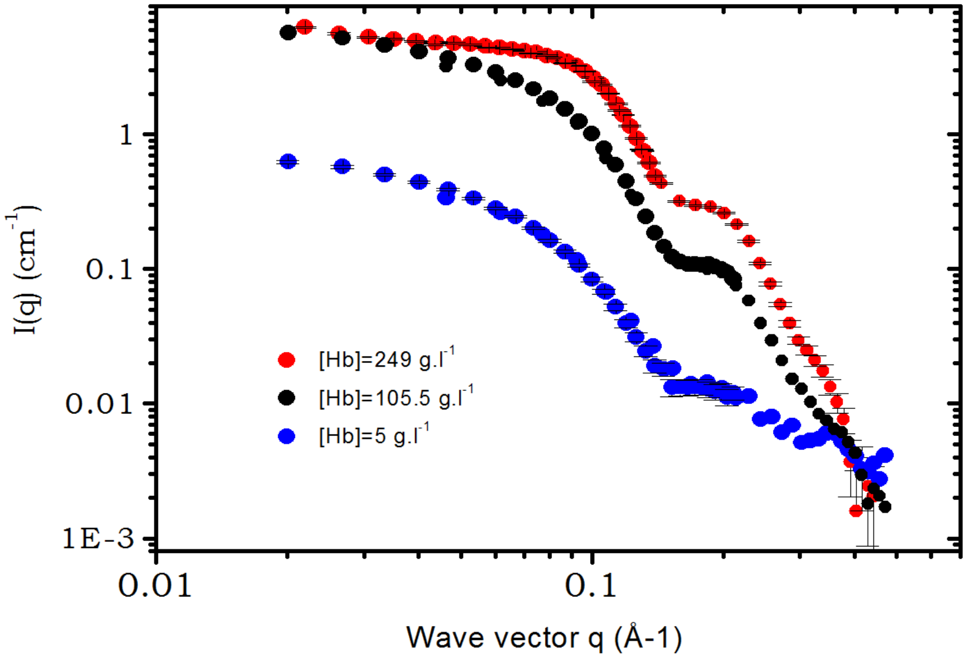

Figure 1: Hemoglobin SANS spectra measured for different protein concentrations, at a temperature of 20°C. At low protein concentration ($5\, g.L^{-1}$) the form factor of the protein is measured. When increasing the concentration up to $\simeq\ 100 g.L^{-1}$ the intensity at q=0, $I(q \to 0)$ increases ($\sim \Phi$) whereas the structure factor remains close to 1 aver the entire wave vector range. At very higher concentration ($\simeq\ 250 g.L^{-1}$) the interaction between proteins become significant and the compressibility of the solution, proportional to $I(q = 0)$ tends to decrease significantly and a correlation peak appears in the structure factor which create a bump in the scattering intensity around $q = 0.1$ Å $^{-1}$. The important point is that there is no significant increase in the scattering intensity at very small wave vector which means that there are no aggregation of the protein in solution. Error bars are smaller than the size of the points.

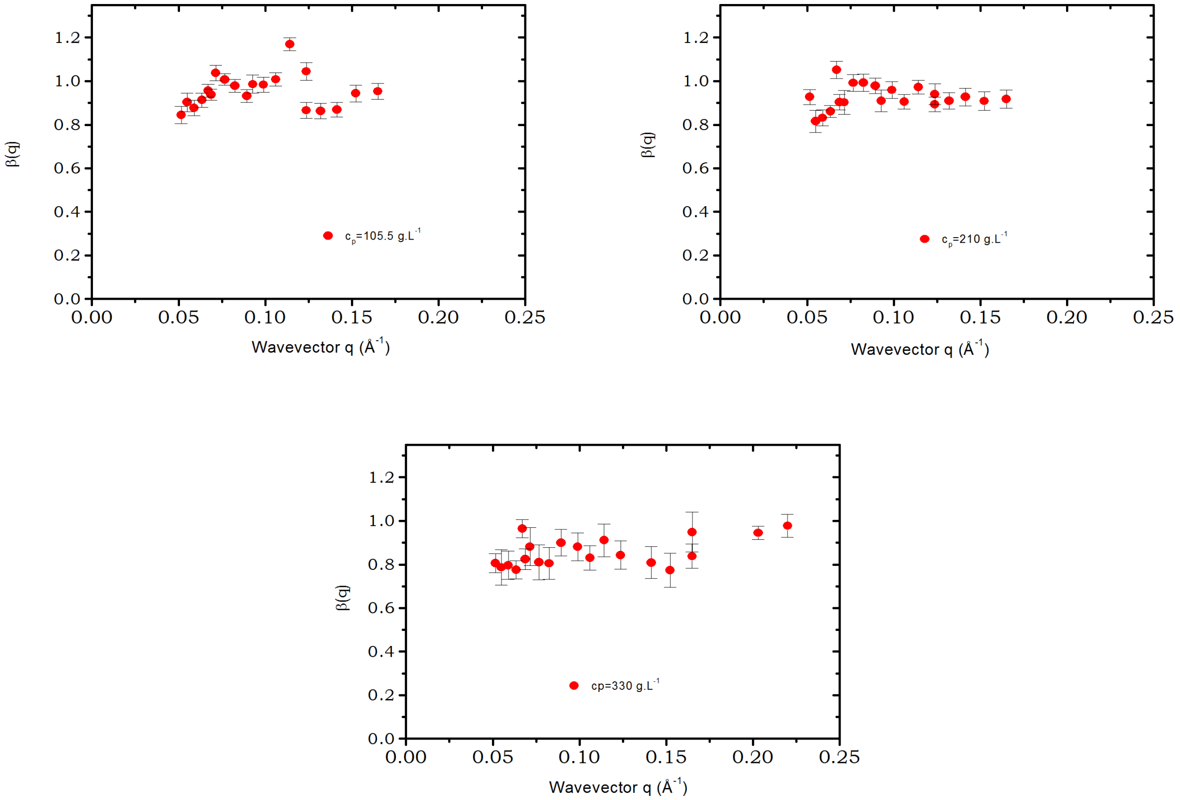

Figure 2: Wave vector dependence of the stretching exponent β of the intermediate scattering function $I(q,t) = e^{-(\frac{t}{\tau})^{\beta}}$ of hemoglobin solution at 3 different concentrations: $c_p = 105.5\, g.L^{-1}$, $c_p = 210\, g.L^{-1}$ and $c_p = 330\, g.L^{-1}$. The self-intermediate scattering function corresponds to the highest q range and show no significant departure from $\beta \simeq 1$, that would be characteristic of an anomalous diffusion.
